# Exploring the Habitat Distribution of *Decapterus macarellus* in the South China Sea Under Varying Spatial Resolutions: A Combined Approach Using Multiple Machine Learning and the MaxEnt Model

**DOI:** 10.3390/biology14070753

**Published:** 2025-06-24

**Authors:** Qikun Shen, Peng Zhang, Xue Feng, Zuozhi Chen, Jiangtao Fan

**Affiliations:** 1 South China Sea Fisheries Research Institute, Chinese Academy of Fishery Sciences, Guangzhou 510300, China; shen_qikun@163.com (Q.S.); zhangpeng@scsfri.ac.cn (P.Z.);; 2 College of Marine Living Resource Sciences and Management, Shanghai Ocean University, Shanghai 201306, China; 3 Key Laboratory for Sustainable Utilization of Open-Sea Fishery, Ministry of Agriculture and Rural Affairs, Guangzhou 510300, China; 4 Guangdong Provincial Key Laboratory of Fishery Ecology and Environment, Guangzhou 510300, China

**Keywords:** *Decapterus macarellus*, spatial resolution, machine learning, SHAP, MaxEnt

## Abstract

The selection of environmental variables with different spatial resolutions is a critical factor affecting the accuracy of machine learning-based fishery forecasting. In this study, spring-season survey data of *Decapterus macarellus* in the South China Sea from 2016 to 2024 were used to construct six machine learning models—decision tree (DT), extra trees (ETs), K-Nearest Neighbors (KNN), light gradient boosting machine (LGBM), random forest (RF), and extreme gradient boosting (XGB)—based on seven environmental variables (e.g., sea surface temperature (SST), chlorophyll-a concentration (CHL)) at four spatial resolutions (0.083°, 0.25°, 0.5°, and 1°), filtered using Pearson correlation analysis. Optimal models were selected under each resolution through performance comparison. SHapley Additive exPlanations (SHAP) values were employed to interpret the contribution of environmental predictors, and the maximum entropy (MaxEnt) model was used to perform habitat suitability mapping. Results showed that the XGB model at 0.083° resolution achieved the best performance, with the area under the receiver operating characteristic curve (ROC_AUC) = 0.836, accuracy = 0.793, and negative predictive value = 0.862, outperforming models at coarser resolutions. CHL was identified as the most influential variable, showing high importance in both the SHAP distribution and the cumulative area under the curve contribution. Predicted suitable habitats were mainly located in the northern and central-southern South China Sea, with the latter covering a broader area. This study is the first to systematically evaluate the impact of spatial resolution on environmental variable selection in machine learning models, integrating SHAP-based interpretability with MaxEnt modeling to achieve reliable habitat suitability prediction, offering valuable insights for fishery forecasting in the South China Sea.

## Simple Summary

In this study, a combined approach integrating multiple machine learning algorithms with the MaxEnt model was applied to systematically evaluate the habitat suitability prediction of *D. macarellus* in the South China Sea under different spatial resolutions. The SHAP method was employed to interpret the contributions of environmental variables within the machine learning framework. The results demonstrated that higher predictive performance was achieved at the finer 0.083° resolution (ROC_AUC = 0.836, accuracy = 0.793, and NPV = 0.862). Furthermore, external validation confirmed that the XGB model exhibited the best overall predictive accuracy and stability, with AUC values approaching 0.9. SHAP analysis identified CHL and SST as the key drivers influencing the distribution of *D. macarellus*, emphasizing their ecological significance. MaxEnt modeling further delineated suitable habitat areas, primarily located in the northern and central-southern regions of the South China Sea. Through comparative analysis of different spatial resolutions and modeling approaches, this study highlights that the combination of 0.083° environmental data and the XGB model is more suitable for investigating the distribution patterns of *D. macarellus* in the South China Sea. These advancements will provide a stronger scientific basis for the sustainable development and management of offshore fishery resources.

## 1. Introduction

Marine ecosystems and fisheries are vital components of human development, and China’s marine fisheries are among the most sensitive to climate change globally [1,2]. Significant shifts in oceanographic conditions have profoundly affected the distribution of fish populations, making accurate fishery forecasting critically important for the sustainable development and effective management of marine fisheries [3,4]. In recent years, remotely sensed oceanographic data with multiple spatial resolutions have been widely used in marine habitat suitability modeling and fishery forecasting [5,6,7]. Given the vastness of the world’s oceans and the logistical and economic constraints of conducting in situ surveys, remote sensing has become an indispensable tool for marine scientists and resource managers. These datasets provide continuous, large-scale, and near-real-time information on key environmental parameters such as SST, CHL, salinity, and ocean currents, which are critical for understanding species–environment interactions. In 2023, based on 0.25° spatial resolution, the spatiotemporal variation in suitable habitats of Sardinops sagax was predicted [8]. In 2023, the habitat distribution of 12 highly migratory top predator species was predicted based on environmental variables at a 0.083° spatial resolution [9]. Exploring the relationship between environmental factors and species habitat distribution, and accurately inferring habitat suitability, play a critical role in promoting sustainable fisheries and conserving marine resources.

The South China Sea (SCS), located at the western edge of the Pacific Ocean, is one of the most important fishing grounds in the world. It is highly vulnerable to climate change, including monsoon-driven circulation, extreme weather events, and long-term oceanographic shifts [10]. Its dynamic ocean environment and rich nutrient conditions support high levels of primary productivity, providing abundant food resources for marine organisms [11,12]. This, in turn, offers favorable habitats for economically important species such as carangids and tunas. The South China Sea contributes a significant proportion of China’s total marine fishery output and serves as a crucial livelihood source for both coastal and distant-water fishers [13]. In recent years, the region has experienced growing ecological pressures from global warming, sea-level rise, intensified human activities, and increasingly frequent extreme climatic events [14,15]. Environmental phenomena such as rising sea surface temperatures and ocean acidification have had profound impacts on marine ecosystems. Since most marine fish migrate toward environments that meet their survival needs, shifts in oceanographic conditions directly affect their distribution patterns. One of the most prominent indicators of ecosystem degradation in the South China Sea is coral reef decline [16]. Coral reefs, as critical components of the marine ecosystem, offer essential habitat for a wide range of fish and invertebrates [17]. However, widespread coral bleaching and mortality driven by climate change have forced many species to seek new, more suitable habitats [18]. These environmental changes not only threaten the stability of fishery yields but also have far-reaching implications for marine biodiversity and habitat distribution. Therefore, accurately identifying the relationship between species habitat distribution and environmental suitability is essential for promoting species sustainability and effective resource management.

Mackerel scad (*Decapterus macarellus*), a newly targeted carangid species in the South China Sea, has emerged as an economically important pelagic fish in recent years [19]. It typically inhabits depths ranging from 40 to 200 m and is widely distributed across tropical and subtropical oceanic regions. However, the stability of its habitat has been increasingly challenged by the impacts of climate change and intensified human activities, which have disrupted marine ecosystems and altered species distributions [20]. At present, research on *D. macarellus* has primarily focused on its biological [21,22,23]. Habitat-related studies have largely relied on species distribution models for prediction [24], yet few have examined how different spatial resolutions of environmental data influence modeling accuracy. Moreover, there is a lack of integrated approaches that combine multiple machine learning algorithms to improve predictive performance and ecological insight. Developing accurate predictive models can significantly enhance the identification of potential fishing and non-fishing grounds, reduce the cost of fishery operations, and improve management efficiency [25,26,27]. Therefore, selecting appropriate spatial resolutions for environmental variables and applying robust modeling approaches are essential to effectively analyze the relationship between *D. macarellus* and its surrounding oceanographic environment. This, in turn, provides a valuable foundation for advancing sustainable fisheries management and resource conservation strategies for *D. macarellus* in the South China Sea.

Fish growth and development are closely related to surrounding oceanic conditions, and habitat prediction based on various environmental factors has become a common approach in contemporary habitat modeling. Early species distributions were primarily based on statistical techniques such as generalized linear models (GLMs) and generalized additive models (GAMs), which were used to analyze the relationships between species presence and environmental variables [28,29,30]. With the development of species distribution, predictive modeling of species habitats has increasingly adopted more advanced techniques, including the MaxEnt model and ecological niche factor analysis (ENFA), both of which have been widely applied to forecast species distributions [31]. Machine learning, which is capable of identifying complex patterns in data and making accurate predictions, has seen rapid advancement in recent years, particularly in the analysis of relationships between fishery resources and environmental drivers [32]. Compared with traditional methods, machine learning algorithms offer improved flexibility and predictive accuracy, especially as data availability and algorithmic sophistication continue to improve. Among these, the RF algorithm—originally proposed by Leo Breiman—is a widely used ensemble learning method [33]. It generates outputs by averaging the predictions of multiple decision trees and is particularly effective in handling uncertainties within complex ecological systems. In addition, several representative machine learning models—including DT, ET, KNN, XGB, and LGBM—have demonstrated significant advantages in capturing complex nonlinear relationships between environmental variables and species distributions. The DT model is a classical machine learning algorithm known for its strong capability in regression and classification tasks [34]. ET is an ensemble learning method based on decision trees and can be considered a variant of the random forest algorithm; it introduces greater randomness during tree construction, thereby improving the model’s generalization ability [35]. KNN is a non-parametric, instance-based supervised learning algorithm commonly used for both classification and regression problems [36]. XGB is an optimized implementation of gradient boosting decision trees (GBDTs), designed to construct a strong ensemble by integrating multiple weak learners, and is suitable for various machine learning tasks, including classification, regression, and ranking [37]. LGBM is also a decision tree–based gradient boosting framework that has been successfully applied across multiple domains due to its efficiency and high scalability [38].

In this study, multiple machine learning algorithms were applied to predict the distribution of *D. macarellus* in the South China Sea using environmental variables at different spatial resolutions. These included the commonly used resolutions of 0.083° and 0.25°, as well as the less frequently utilized 0.5° and 1°. Based on the performance metrics of each model, the optimal algorithm was selected for each spatial resolution. Habitat suitability visualization was subsequently conducted using the MaxEnt model. The contribution and influence of each environmental variable were further interpreted using SHAP values and cumulative area under the curve (AUC) plots. The objective of this study was to identify the most suitable spatial resolution and the best-performing model for predicting the habitat of *D. macarellus* in the South China Sea. The findings aim to provide a scientific basis for the development and management of *D. macarellus* fishery resources in the region.

## 2. Materials and Methods

### 2.1. Sampling Information of D. macarellus

**Figure 1:**
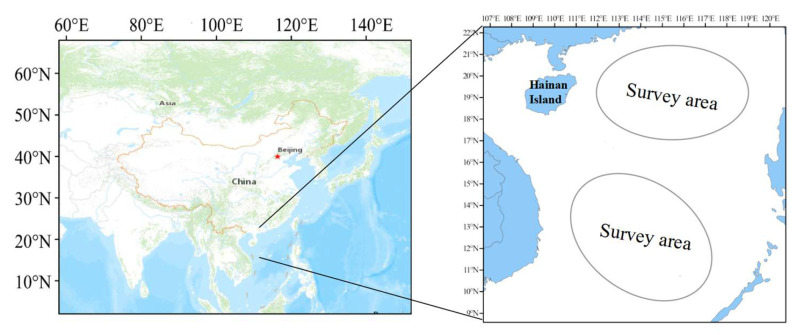
Maps showing the area where the study fish were sampled.

This study utilized spring survey data of *D. macarellus* collected from the South China Sea during the 2016–2022 period for model training, while data from 2023 to 2024 were used as an external validation dataset. The surveys were conducted using a commercial fishing vessel equipped with a main engine rated at 441 kW. The vessel measured 36.8 m in length, 6.8 m in width, and had a draft of 3.8 m. The sampling area covered the northern and central-southern regions of the South China Sea (Figure 1). The trawl net used for these surveys measured 76 m × 53.79 m × 34 m, with a mesh size of 200 mm and a cod-end mesh size of 39 mm. In most cases, sampling was conducted three times per day, with the net towed at an average speed of 3.5 knots for one hour. However, in certain nearshore areas and fishing grounds, the towing duration was reduced to 30–50 min to avoid collisions with other vessels or fixed fishing gear. Within the study area, sampling sites with a high occurrence frequency of *D. macarellus* were selected. These sites exhibited relatively high biomass, supporting their significance in habitat identification. Ultimately, 117 distinct and validated sampling locations were chosen for analysis.

### 2.2. Screening of Environmental Data

For the selection of environmental variables, seven key parameters (SSS, SSH, MLD, SST, DIS, BATH, and CHL) were obtained from the Copernicus Marine Service (https://marine.copernicus.eu/ accessed on 10 April 2025) and the Global Fishing Watch System (https://globalfishingwatch.org/ accessed on 10 April 2025) (Table 1). The selection of these parameters was based on their recognized importance in influencing the distribution of marine species. To prevent model overfitting and ensure accurate model evaluation, Pearson correlation analysis was conducted on the seven selected environmental variables to avoid excessive multicollinearity and intercorrelation among predictors. To reduce interdependence among environmental factors, only variables with Pearson correlation coefficients below 0.8 (|R| < 0.8) were retained, where values close to −1 or 1 indicate strong negative or positive correlations, respectively. Environmental data were downloaded at spatial resolutions of 0.083° and 0.25°, while datasets originally at 0.5° and 1° resolution were resampled and extracted using ArcGIS 10.7.

### 2.3. Model Construction and Performance Evaluation

Machine learning offers strong nonlinear fitting capabilities and can automatically extract features from complex, high-dimensional data, thereby improving predictive accuracy. It also demonstrates high flexibility and efficiency in handling large datasets and variable selection. In this study, six machine learning algorithms—decision tree (DT), extra trees (ETs), K-nearest neighbors (KNN), light gradient boosting machine (LGBM), random forest (RF), and extreme gradient boosting (XGB)—were employed to train predictive models. Model performance was evaluated through comparative analysis, and the optimal algorithm was selected for further application. Following the selection of the optimal model, SHAP values and cumulative AUC curves were employed to assess both the relative importance and the directional influence of environmental factors on species distribution. Based on this, species habitat suitability was predicted using the MaxEnt model. By comparing different machine learning models, this study aimed to enhance the accuracy and robustness of species distribution predictions under varying environmental conditions. All modeling procedures were implemented in R. In species distribution modeling, it is essential to compare environmental conditions with known species occurrences, which requires a sufficient number of presence records. However, due to geographical and economic constraints, actual sampling data for most species are often limited. As a result, the use of pseudo-absence data as a substitute has become a widely accepted approach in model construction. In this study, pseudo-absence points were randomly generated at three times the number of actual presence records within the study area to support model construction [39]. Overlapping points were removed to ensure spatial independence among all locations, thereby enhancing the predictive accuracy of the model. During the modeling process, a cross-validation approach was employed. The dataset was randomly split into the following two subsets: 70% for model training and 30% for validation. To ensure robustness, the modeling procedure was repeated 5 times. Model performance was evaluated using the following five metrics: negative predictive value (NPV), positive predictive value (PPV), specificity, area under the receiver operating characteristic curve (ROC_AUC), and accuracy. Generally, an NPV greater than 0.8 indicates good model performance, while a PPV above 0.5 suggests that the model is acceptable. A specificity value exceeding 0.8 also reflects good performance. Similarly, a ROC_AUC value above 0.8 is considered indicative of strong discriminatory ability, and an accuracy higher than 0.8 denotes satisfactory overall model performance [40]. The formulas for calculating each evaluation metric are as follows [41,42]:

(1) $$Accuracy=\frac{TP+TN}{TP+TN+FP+FN}$$

(2) $$Specificity=\frac{TN}{TN+FP}$$

(3) $$PPV=\frac{TP}{TP+FP}$$

(4) $$NPV=\frac{TN}{TN+FN}$$

In these formulas, TP (true positive) refers to correctly predicted presence points, FN (false negative) refers to actual presence points incorrectly predicted as absence, TN (true negative) refers to correctly predicted pseudo-absence points, and FP (false positive) refers to pseudo-absence points incorrectly predicted as presence. AUC is calculated based on the area under the ROC curve, which plots the true positive rate (sensitivity) against the false positive rate (1 − specificity).

### 2.4. Model Parameter Settings

In this study, multiple machine learning algorithms, including DT, ET, KNN, RF, LGBM, and XGB, were employed to predict the habitat suitability of *D. macarellus* in the South China Sea. The key hyperparameters for each model were set as follows: for XGB, the learning rate (eta) was set to 0.1, the maximum depth (max_depth) to 6, and the number of boosting rounds (nrounds) to 100. For RF, the number of trees (ntree) was set to 500, and the number of variables randomly selected at each split (mtry) was optimized through a grid search with tuneLength = 3. The ET model was configured with ntree = 500, mtry = 3, and nodesize = 1. For LGBM, the maximum depth was set to 6, the learning rate to 0.1, the number of trees to 100, and the Bernoulli distribution was used for binary classification tasks. The KNN model was set with k = 5, and feature standardization (centering and scaling) was applied prior to modeling. The DT model was trained using default parameter settings. All models were trained and validated using five-fold cross-validation to optimize hyperparameters and prevent overfitting. The detailed parameter settings for each machine learning model are summarized in Table 2.

## 3. Results

### 3.1. Screening Factors

**Figure 2:**
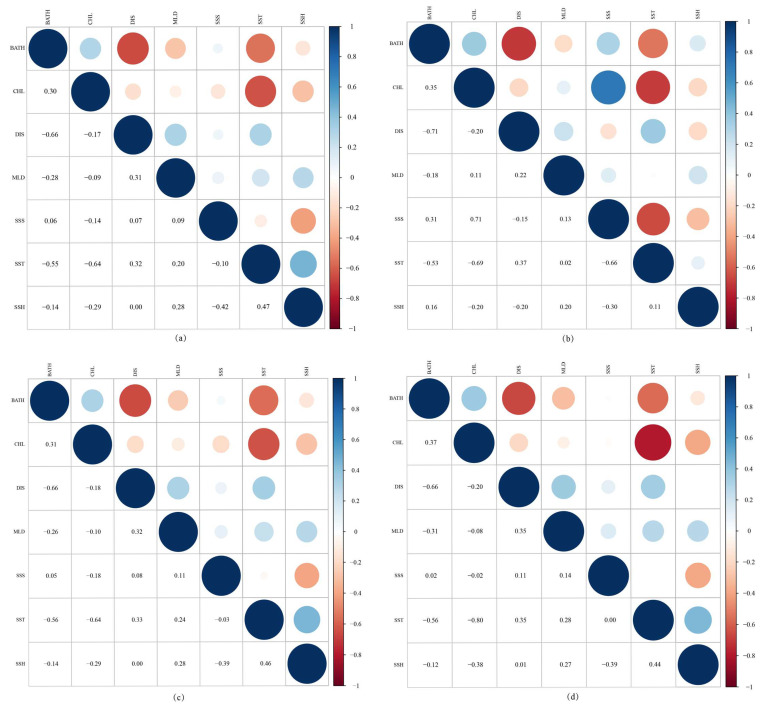
Pearson correlation heatmaps of environmental variables at four spatial resolutions. (BATH: bathymetry, CHL: mass concentration of chlorophyll-a in seawater, DIS: distance from shore, MLD: ocean mixed layer thickness, SSS: seawater salinity, SST: sea surface temperature, SSH: sea surface height above geoid) (**a**) Pearson correlation heatmap of environmental variables at 0.083° spatial resolution; (**b**) Pearson correlation heatmap of environmental variables at 0.25° spatial resolution; (**c**) Pearson correlation heatmap of environmental variables at 0.5° spatial resolution; (**d**) Pearson correlation heatmap of environmental variables at 1° spatial resolution.

Pearson correlation analyses were conducted for environmental datasets at four different spatial resolutions (Figure 2). At a resolution of 0.083° (Figure 2a), the highest correlation was observed between DIS and BATH (r = −0.66), followed by SST and CHL (r = −0.64). At 0.25° resolution (Figure 2b), the strongest correlation was also between DIS and BATH (r = −0.71), followed by SSS and CHL (r = 0.71). At 0.5° resolution (Figure 2c), the highest correlation remained between DIS and BATH (r = −0.66). At 1° resolution (Figure 2d), SST and CHL showed the strongest correlation (r = −0.80). Overall, across all resolutions, the Pearson correlation coefficients among the seven environmental variables were below the threshold of 0.8 (|r| < 0.8), indicating that all variables were suitable for inclusion in the predictive models.

### 3.2. Model Performance

**Figure 3:**
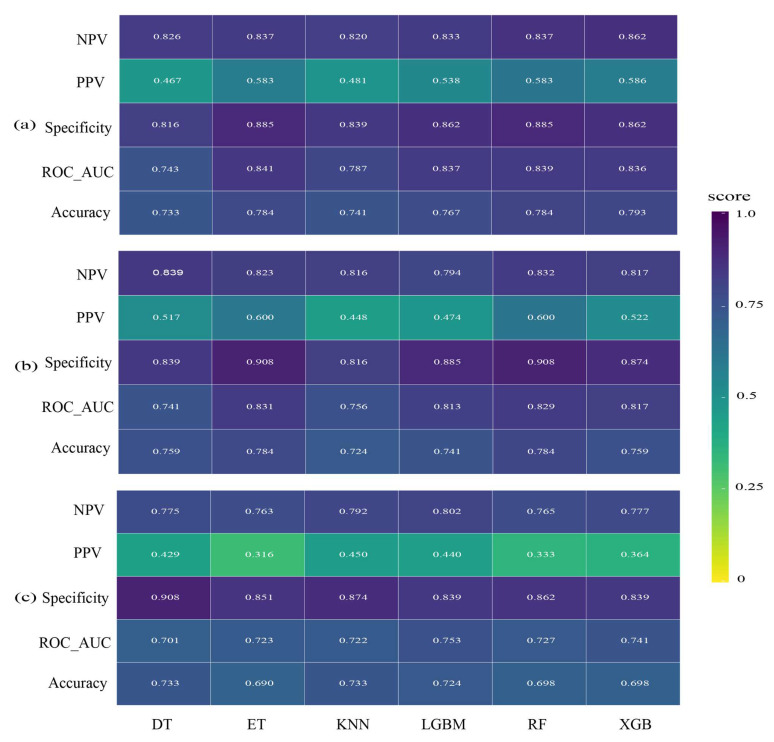
Model performance comparison at different spatial resolutions. (**a**) Model performance comparison at 0.083° spatial resolution; (**b**) model performance comparison at 0.25° spatial resolution; (**c**) model performance comparison at 0.5° spatial resolution.

The selected environmental variables were incorporated into the models at different spatial resolutions, and the corresponding values of NPV, PPV, specificity, ROC_AUC, and accuracy were calculated for each model (Figure 3). Based on a comprehensive comparison of model performance, the XGB model achieved the best results at the 0.083° spatial resolution, with NPV, specificity, and ROC_AUC values all exceeding 0.8, indicating strong predictive capability (Figure 3a). Additionally, the accuracy reached 0.793, which is close to 0.8 and further supports the model’s reliability (Figure 3a). At the 0.25° resolution, the ET model outperformed other models, with a specificity value of 0.908 and both NPV and ROC_AUC above 0.8, suggesting good predictive performance (Figure 3b). In contrast, at the 0.5° resolution, the PPV values ranged between 0.3 and 0.45, indicating a high error rate (over 50%); thus, this resolution was excluded from further analysis (Figure 3c). A 1° resolution was also tested; however, the configuration of pseudo-absence points was not suitable for modeling at this scale, leading to considerable inaccuracies. As a result, data at this resolution were deemed unreliable and excluded from the final model predictions.

By comparing the results, it was found that although the 0.25° spatial resolution yielded a higher specificity value (0.908) compared to that of the 0.083° resolution (0.862), it performed worse in terms of ROC_AUC, accuracy, and NPV. Therefore, the prediction at the 0.083° resolution is considered to have higher overall precision and better predictive performance.

### 3.3. Analysis of Variable Importance

**Figure 4:**
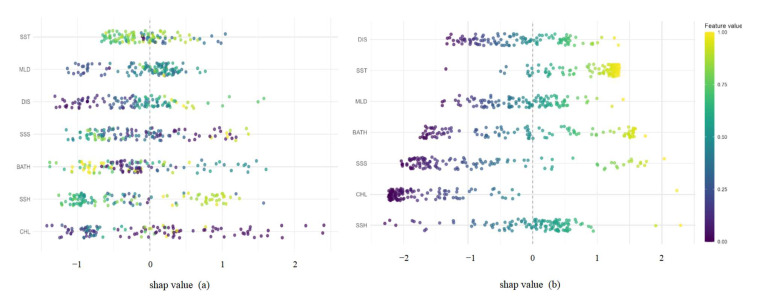
SHAP bee swarm plots of environmental variables at different spatial resolutions. (**a**) SHAP bee swarm plot of environmental variables at 0.083° spatial resolution; (**b**) SHAP bee swarm plot of environmental variables at 0.25° spatial resolution.

**Figure 5:**
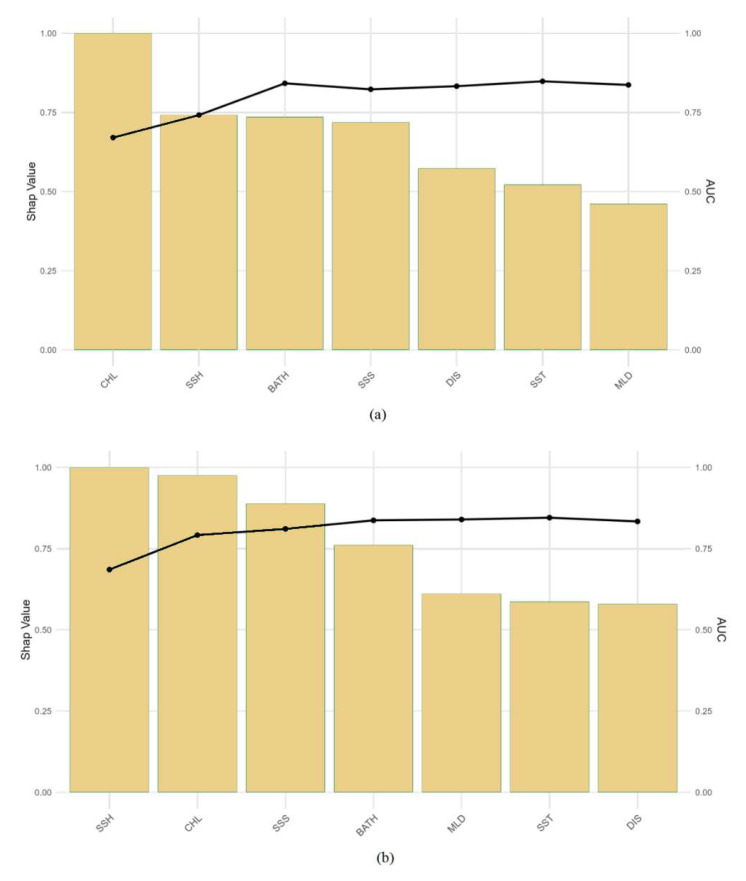
Cumulative AUC plots of environmental variables at different spatial resolutions. (**a**) Cumulative AUC plot of environmental variables at 0.083° spatial resolution; (**b**) cumulative AUC plot of environmental variables at 0.25° spatial resolution.

The SHAP approach was used to quantify the relative importance of environmental variables within the optimal model (Figure 4). Additionally, cumulative AUC plots were employed to visually assess how the sequential inclusion of each environmental factor influenced model accuracy (Figure 5). At the 0.083° spatial resolution, the SHAP value distribution for CHL exhibited a wide range, with high SHAP values predominantly negative, suggesting that elevated chlorophyll-a concentrations may be unfavorable for the habitat suitability of *D. macarellus* in the South China Sea (Figure 4a). SST showed the most pronounced positive influence in the model, with a concentration of high SHAP values, indicating that warmer waters enhance the species’ habitat suitability and that *D. macarellus* tends to prefer warmer environments (Figure 4a). Both DIS and BATH showed positive contributions at intermediate values, suggesting a preference for mid-range environmental gradients (Figure 4a). In contrast, MLD and SSH exhibited no clear directional trend, with highly variable SHAP values, possibly reflecting interactions with other environmental factors (Figure 4a). The overall contribution of SSS was relatively low, with no consistent pattern observed (Figure 4a). At the 0.25° spatial resolution, SST, BATH, and DIS emerged as the most important positive predictors in the model, representing the favorable effects of warm waters, deeper environments, and offshore distance on the distribution of *D. macarellus* (Figure 4b). In contrast, higher concentrations of chlorophyll a (CHL) significantly suppressed the probability of species occurrence, which may be attributed to increased water turbidity or intensified ecological competition resulting from high phytoplankton density (Figure 4b). Other variables, such as MLD, SSH, and SSS, showed relatively minor influence (Figure 4b). Comparative analysis of the results across the two spatial resolutions revealed a consistent pattern: elevated SST positively influenced the habitat suitability of *D. macarellus* in the South China Sea, while high CHL concentrations had a negative impact.

The cumulative AUC plots were used to evaluate the contribution of the seven environmental variables in the predictive models (Figure 5). At the 0.083° spatial resolution, CHL contributed the most to model performance, followed by a noticeable increase after the inclusion of SSH and BATH (Figure 5a). A slight decrease was observed after the addition of SSS, while the inclusion of DIS and SST led to minor increases in AUC (Figure 5a). The curve eventually stabilized after the addition of MLD (Figure 5a). At the 0.25° spatial resolution, SSH exhibited the highest contribution, and a gradual upward trend in AUC was observed as CHL, SSS, BATH, MLD, and SST were sequentially added. The curve plateaued following the inclusion of DIS (Figure 5b). Overall, CHL and SSH were consistently identified as the top two contributing environmental variables at both spatial resolutions. CHL also exhibited high activity in the SHAP value distribution, further highlighting its influence. These findings suggest that CHL is a key environmental factor shaping the habitat suitability and distribution of *D. macarellus* in the South China Sea.

### 3.4. Habitat Suitability Prediction for D. macarellus in the South China Sea

**Figure 6:**
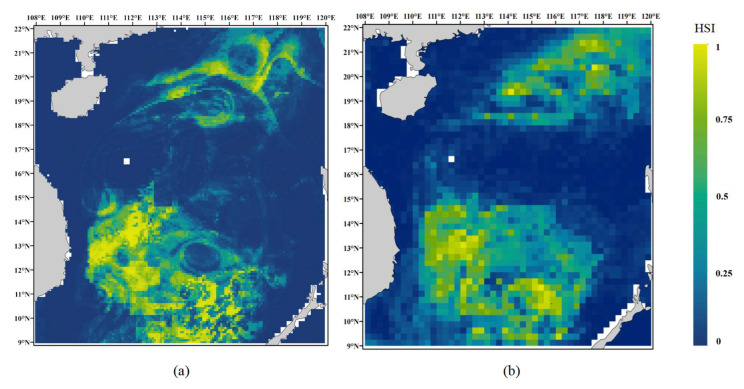
Comparison of *D. macarellus* habitat suitability maps at multiple resolutions in the South China Sea. (**a**) Habitat suitability map of *D. macarellus* at 0.083° spatial resolution; (**b**) habitat suitability map of *D*. *macarellus* at 0.25° spatial resolution.

Machine learning models were trained and used for prediction based on true presence data from 2016 to 2022 and artificially generated pseudo-absence points. After selecting the optimal models through performance comparison, habitat suitability predictions were conducted using the MaxEnt model. The XGB model was identified as the best-performing algorithm at the 0.083° spatial resolution, while the ET model showed the highest performance at the 0.25° resolution. The prediction results from the two spatial resolutions showed a high degree of similarity, both indicating that the suitable habitat of *D. macarellus* in the South China Sea is primarily located in the northern and central-southern regions, with the latter covering a larger area. At the 0.083° resolution, the higher spatial precision allowed for clearer visualization of suitable habitats, which were mainly concentrated in the central-southern South China Sea, particularly between 9–15° N and 110–117° E (Figure 6). Although the map at 0.25° resolution appeared less clear due to a coarser grid size, the core suitable habitat areas closely matched those observed at the 0.083° resolution (Figure 6b). Based on the model evaluation metrics, the 0.083° spatial resolution yielded higher predictive accuracy compared to the 0.25° resolution. In conjunction with the habitat suitability maps, it was evident that higher spatial resolution resulted in more precise identification of suitable areas (Figure 6a).

### 3.5. External Validation

**Figure 7:**
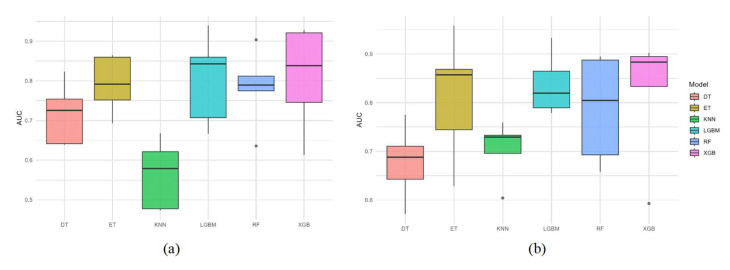
AUC comparison across models using the external dataset. (**a**) Cross-model AUC values at 0.083° spatial resolution using an external dataset; (**b**) cross-model AUC values at 0.25° spatial resolution using an external dataset.

**Figure 8:**
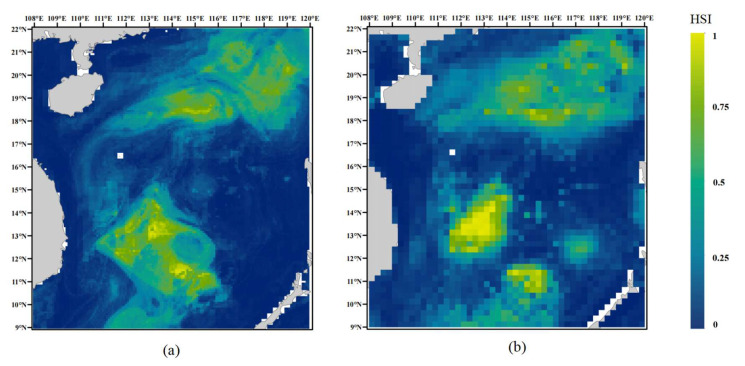
Habitat suitability map of *D. macarellus* based on the best model using the external dataset. (**a**) Habitat suitability map of *D. macarellus* at 0.083° spatial resolution using the best model based on the external dataset; (**b**) habitat suitability map of *D. macarellus* at 0.25° spatial resolution using the best model based on the external dataset.

Using 2023–2024 survey data as an external validation dataset, multiple machine learning models were compared at two spatial resolutions (0.083° and 0.25°). The results showed that the XGB model outperformed the other models at both resolutions, with AUC values approaching 0.9 (Figure 7). In addition, a comparison of the XGBoost model’s performance across different spatial resolutions revealed that its predictive stability was stronger at the 0.083° resolution (Figure 7a) than at 0.25° (Figure 7b). Habitat suitability predictions generated using the optimal model revealed that the suitable habitat of *D. macarellus* was primarily located in the northern and central-southern regions of the South China Sea, consistent with the findings from the training dataset (Figure 8). This external validation supports the robustness and high predictive accuracy of the model, confirming the reliability of the predicted distribution patterns. Therefore, we conclude that the 0.083° resolution is more appropriate for predicting the habitat suitability of *D. macarellus* in the South China Sea using a combined approach of machine learning and the MaxEnt model.

## 4. Discussion

### 4.1. Justification for the Selection of Environmental Variables

Marine environmental factors play a crucial role in shaping the distribution of *D. macarellus* in the South China Sea. Among them, CHL influences fishery grounds through its role in the marine food chain, as it reflects the abundance of phytoplankton—the primary producers supporting higher trophic levels [43]. SST is one of the most fundamental environmental parameters affecting fish behavior, metabolism, and distribution [44]. For pelagic species like *D. macarellus*, DIS and BATH are essential spatial variables that influence habitat accessibility and depth preference [45]. SSS is particularly important for migratory species, as salinity gradients often guide movement patterns [46]. SSH, which integrates oceanographic features such as currents, thermal structures, salinity fronts, and eddies, is commonly used in fishery analysis to identify dynamic oceanographic zones favorable for fish aggregation. In this study, a total of seven environmental variables—including CHL, SST, and SSH—were selected to improve the model’s capacity to capture the ecological and spatial preferences of *D. macarellus*. Our results identified CHL as a key factor influencing the species’ distribution. The SHAP analysis indicated that higher CHL concentrations were associated with a negative contribution to habitat suitability for *D. macarellus*. Biologically, this result may be explained by the species’ preference for offshore pelagic environments with relatively moderate primary productivity, rather than eutrophic nearshore waters. As an indicator of phytoplankton biomass, CHL is closely linked to feeding availability, which is essential for the survival and aggregation of *D. macarellus* [47]. High CHL concentrations typically occur in coastal areas with strong nutrient input and higher phytoplankton density, which may coincide with lower oxygen levels, increased turbidity, and anthropogenic stressors—conditions less favorable for this species. As a fast-swimming, migratory, mid-trophic level fish, *D. macarellus* tends to aggregate in mesotrophic areas where prey (e.g., zooplankton, small pelagic forage) is abundant but not over-concentrated. Spring is the main spawning season for *D. macarellus* in the South China Sea, and pre-spawning feeding behavior may be the primary reason why CHL emerged as a key influencing factor [48]. These findings are consistent with previous studies using SDM models to assess the species’ distribution in the South China Sea [24]. The results of this study indicate that *D. macarellus* prefers oceanic, warm-water ecosystems, where elevated sea surface temperatures and abundant zooplankton resources create favorable conditions for foraging and group migration. Overall, *D. macarellus* tends to inhabit offshore areas characterized by higher temperatures, moderate distances from the coast, and intermediate depths, reflecting the typical ecological traits of pelagic oceanic species. In this study, the application of the SHAP method provided significant advantages in interpreting the contributions of environmental factors. Unlike traditional variable importance measures, SHAP values quantify both the magnitude and the direction (positive or negative) of each factor’s contribution to the predictions, enabling a more transparent understanding of how environmental drivers influence species distribution [49]. The results revealed that key drivers such as CHL and SST played important roles in modulating the habitat suitability of *D. macarellus* in the South China Sea. A major advantage of SHAP lies in its ability to link machine learning model outputs to biological mechanisms, offering clearer explanations of the positive or negative effects of environmental variables on species predictions [50]. This approach enhances both the interpretability of the models and their ecological relevance.

### 4.2. Model Performance Comparison

In this study, the XGB model achieved the best predictive performance at the 0.083° spatial resolution, with ROC_AUC = 0.836, accuracy = 0.793, and NPV = 0.862, outperforming the ET model, which was optimal at the 0.25° resolution. Furthermore, in external validation using the 2023–2024 dataset, XGB remained the best-performing model across both spatial resolutions (0.083° and 0.25°), with AUC values approaching 0.9, demonstrating outstanding predictive robustness. XGB constructs an ensemble of decision trees sequentially, with each new tree specifically trained to correct the residual errors of the previous ensemble [51]. This approach enables the model to more effectively capture the complex nonlinear relationships between environmental factors and species distribution. A core advantage of XGB lies in its use of both first-order (gradient) and second-order (Hessian) derivatives during model training [52]. By accurately estimating the curvature of the loss function, XGBoost achieves more precise and stable updates at each iteration, which is critical for ecological datasets where environmental variables are often highly nonlinear and where small improvements can significantly enhance model performance. Additionally, XGB’s flexibility in modeling complex patterns, its strong regularization mechanisms, and its ability to mitigate overfitting collectively explain why it consistently outperformed other machine learning models across different spatial resolutions in this study.

### 4.3. Comparison of Spatial Resolution Effects

The comparison across different spatial resolutions clearly demonstrated that the resolution of environmental data has a significant impact on habitat suitability prediction outcomes [53]. Models constructed using 0.083° resolution data achieved higher predictive accuracy than those based on 0.25°, and were markedly superior to models using 0.5° or 1° resolution data. Specifically, at 0.083° resolution, the XGB model exhibited the highest ROC_AUC, accuracy, and NPV compared to other resolutions, although the 0.25° models also demonstrated a certain level of reliability. External validation further confirmed that at both 0.083° and 0.25° resolutions, XGBoost remained the optimal model; however, model stability was notably higher at 0.083°. The superior performance and stability at finer resolutions are likely attributable to the enhanced ability to capture small-scale environmental heterogeneity, which is critical for the habitat selection of *D. macarellus* in the dynamic South China Sea. In contrast, coarser resolutions tend to excessively smooth local variability, masking important microhabitat features necessary for accurate modeling. Additionally, environmental variables are undergoing subtle changes under the influence of climate change, and such fine-scale variations are more easily detected using high-resolution data. Compared to highly migratory species such as tunas [54,55], where 0.5° or 1° resolutions are commonly used for fishery forecasting [56,57], the relatively localized movement patterns of *D. macarellus* make the 0.083° resolution more suitable for spatial distribution analysis. A study on the distribution of Caspian kutum (Rutilus frisii) indicated that higher spatial resolutions yielded stronger model accuracy, whereas coarser spatial resolutions could lead to reduced predictive reliability and model performance [58]. Studies exploring the effects of spatial scale on fish habitat modeling have shown that models based on higher spatial resolutions generally achieve better predictive performance [59]. Research investigating different spatial scales produced results consistent with those of the present study, thereby supporting the validity and robustness of our findings. High-resolution environmental data allow for more precise detection of habitat shifts and ecological responses, thereby providing a better basis for effective management and conservation strategies [60,61,62].

### 4.4. Ecological Rationality of Predicted Potential Habitats

The MaxEnt model is widely used for habitat suitability prediction due to its advantage of requiring only species presence data, making it particularly suitable for marine ecosystems where data collection is often challenging or incomplete [63]. While machine learning models (XGB, RF, ET. e.g.) are highly effective in capturing complex nonlinear relationships between multiple environmental variables and species distributions, they often lack the intuitive spatial visualization capabilities necessary for management applications. MaxEnt, grounded in ecological niche theory, complements this limitation by generating clear, spatially explicit habitat suitability maps [64,65]. Results from both internal and external validations in this study showed that the suitable habitats for *D. macarellus* were mainly located in the northern and central-southern regions of the South China Sea, characterized by favorable sea surface temperatures, moderate depths, and intermediate distances from the coast. These patterns are consistent with the ecological traits of *D. macarellus* as an epipelagic, oceanic species. Regionally, the distribution suggests the potential for broad-scale migratory behavior; however, further research is needed to confirm the extent of its migratory patterns. Given the increasing pressures from climate change and human activities, accurately identifying suitable habitats and delineating potential fishing grounds will be critical for reducing search costs in fisheries operations and supporting sustainable fishery resource management [66]. The integration of MaxEnt habitat modeling with machine learning-based prediction offers a powerful framework to meet these challenges in dynamic marine ecosystems like the South China Sea.

## 5. Conclusions

In this study, a combined approach integrating multiple machine learning algorithms with the MaxEnt model was applied to systematically evaluate the habitat suitability prediction of *D. macarellus* in the South China Sea under different spatial resolutions. The SHAP method was employed to interpret the contributions of environmental variables within the machine learning framework. The results demonstrated that higher predictive performance was achieved at the finer 0.083° resolution (ROC_AUC = 0.836, accuracy = 0.793, and NPV = 0.862). Furthermore, external validation confirmed that the XGB model exhibited the best overall predictive accuracy and stability, with AUC values approaching 0.9. SHAP analysis identified CHL and SST as the key drivers influencing the distribution of *D. macarellus*, emphasizing their ecological significance. MaxEnt modeling further delineated suitable habitat areas, primarily located in the northern and central-southern regions of the South China Sea. Through comparative analysis of different spatial resolutions and modeling approaches, this study highlights that the combination of 0.083° environmental data and the XGB model is more suitable for investigating the distribution patterns of *D. macarellus* in the South China Sea. Future research should explore the effects of additional environmental factors, model parameter optimization, and the integration of oceanographic model outputs to further enhance prediction accuracy and practical applicability. These advancements will provide a stronger scientific basis for the sustainable development and management of offshore fishery resources.

## Figures and Tables

**Table 1:** Candidate factors and data sources.

Variable	Description	Source	Unit	Spatial Resolution
SSS	Seawater salinity	https://marine.copernicus.eu/	‰	0.083° and 0.25°
SSH	Sea surface height above geoid	https://marine.copernicus.eu/	m	0.083° and 0.25°
MLD	Ocean mixed layer thickness	https://marine.copernicus.eu/	m	0.083° and 0.25°
SST	Sea surface temperature	https://marine.copernicus.eu/	°C	0.083° and 0.25°
DIS	Distance from shore	https://globalfishingwatch.org/	km	0.083°
BATH	Bathymetry	https://globalfishingwatch.org/	m	0.083°
CHL	Mass concentration of chlorophyll-a in seawater	https://marine.copernicus.eu/	mg⋅m^−3^	0.25°

**Table 2:** Parameter settings of the model.

Models	Parameter Settings
RF	tuneLength = 3; ntree = 500
DT	rpart
XGB	Eta = 0.1; max_depth = 0.8; nrounds = 100
KNN	K = 5
ET	ntree = 500; mtry = 3; nodesize = 1
LGBM	distribution = “bernoulli”; n.trees = 100; interaction.depth = 6; shrinkage = 0.1

## Data Availability

Data will be made available upon request.
